# The Role of KRAS Mutational Analysis to Determine the Site of Origin of Metastatic Carcinoma to the Lung: A Case Report

**DOI:** 10.1155/2012/425967

**Published:** 2012-10-17

**Authors:** Ahmed N. Alkhasawneh, Hui-Jia Dong, Chen Liu, Carmen Allegra, Robert W. Allan

**Affiliations:** ^1^Department of Pathology, Immunology, and Laboratory Medicine, College of Medicine, University of Florida, P.O. Box 100275, 1600 SW Archer Road, Gainesville, FL 32610, USA; ^2^Division of Hematology and Oncology, College of Medicine, University of Florida, 1600 SW Archer Road, Gainesville, FL 32610, USA

## Abstract

Metastatic carcinomas involving the lung are a common specimen encountered in surgical pathology. These metastases may have different morphologic, and architectural patterns and may mimic primary pulmonary adenocarcinoma, especially the intra-alveolar (lepidic) pattern of spread which may simulate a primary pulmonary bronchioloalveolar carcinoma (adenocarcinoma in situ). We present the case of a metastatic pancreatic adenocarcinoma that morphologically mimicked bronchioloalveolar carcinoma of the lung in that the tumor had an exclusive intra-alveolar pattern of spread and had an immunophenotype that was noninformative as to the site of origin (cytokeratin 7+, cytokeratin 20−, TTF-1−). In this case, we used KRAS gene mutation analysis to support that the lung carcinoma represented a metastatic pancreatic carcinoma as they both possessed identical codon 12 KRAS mutations. We show that this method may be a useful way to prove site of origin of metastatic carcinoma—particularly if standard morphologic or immunohistochemical analysis is not definitive.

## 1. Introduction

The lungs are a frequent site of both metastatic and primary carcinoma. Many different patterns of metastases to the lungs have been described: nodules/masses, lymphangitic carcinomatosis, tumor emboli, endobronchial growth, and intra-alveolar (lepidic) spread. In intra-alveolar spread, the tumor cells replace the lining of alveoli, mimicking bronchioloalveolar carcinoma (adenocarcinoma in situ). Metastases from gastrointestinal tract carcinomas, including the pancreas, are known to have an intra-alveolar pattern of spread [1, 2]. Immunohistochemical studies are usually helpful in distinguishing between these entities. For example, if the tumor expresses thyroid transcription factor-1 (TTF-1) this is strong evidence that the tumor is a pulmonary primary. However, in many instances immunohistochemistry may not be as useful, particularly when one encounters an adenocarcinoma with mucinous differentiation that is TTF-1 negative. In this setting, morphology and immunohistochemistry may not be definitive and clinical correlation is often relied on to make the distinction.

Mutations involving the Kirsten rat sarcoma viral oncogene homolog (KRAS) gene are present in the vast majority of pancreatic adenocarcinomas (more than 90%) and less frequently in pulmonary adenocarcinoma (15–30%) [3–6]. In this paper, we used the identification of a KRAS mutation as a molecular signature of a metastatic pancreatic adenocarcinoma to the lung and thus show that this technique can be used to identify site of origin of metastatic carcinoma.

## 2. Case Report

A 60-year-old male with a medical history of coronary artery disease, abdominal aortic aneurysm, and 60 pack-year smoking history developed painless jaundice one year before evaluation. His CA 19.9 was elevated at 2770 U/mL (reference interval 0–25 U/mL). Computed tomography (CT) of the chest, abdomen, and pelvis showed a 3.0 cm mass involving the head of the pancreas associated with dilation of the main pancreatic duct. Multiple small nodules were noted in the lungs that initially were felt to represent pneumonia; antibiotics were administered. One month after presentation the patient underwent laparotomy for his pancreatic mass. The lung lesions noted on CT scan persisted and the patient underwent CT guided lung biopsy.

## 3. Materials and Methods

### 3.1. Histology and Immunohistochemistry

Standard hematoxylin and eosin (H&E) stained sections of the pathologic specimens were examined. The immunohistochemical studies were performed at our hospital laboratories. These included thyroid transcription factor-1 (TTF-1, 8G7G3/1 clone, Cell Marque, Rocklin, CA, USA), cytokeratin 7 (OV-TL clone, Dako, Carpinteria, CA, USA), and cytokeratin 20 (K520.8 clone, Dako, Carpinteria, CA, USA). The immunostaining was performed on the Ventana BenchMark XT (Ventana Medical Systems, Tucson, AZ, USA) using the standard procedures per the manufacturer's instructions. Hematoxylin counterstain was used.

### 3.2. DNA Extraction and PCR Amplification

Manual dissection of regions containing greater than 50% tumor was performed from the formalin-fixed, paraffin-embedded (FFPE) block. DNA extraction was performed according to the manufacturer's protocol (Trimgen WAXFREE paraffin DNA extraction kit, Sparks, MD, USA). Polymerase chain reaction (PCR) was performed on a GeneAmp PCR system 9700. The primers used to amplify the KRAS gene codon 12 were Biotin labeled forward PCR primers 5′-biotin-TGACTGAATATAAACTTGTGGTAGTTG-3′ and reverse primer 5′-TCGTCCACAAAATGATTCTGAA-3′. The sequence primers were 12p1: 5′-GCA CTC TTG CCT ACG CCA C, 12p2: 5′-GCA CTC TTG CCT ACG CCA, and 13p2: 5′-GCA CTC TTG CCT ACG. The PCR program was as follows: 95°C 5 minutes; 95°C for 20 seconds, 58°C, hold for 30 seconds, 72°C, hold for 20 seconds; for 40 cycles, 72°C for 5 minutes.

### 3.3. Detection of KRAS Mutations by Pyrosequencing

A 20 uL of biotinylated PCR product was immobilized on streptavidin coated Sepharose beads (Streptavidin Sepharose High Performance, GE Healthcare, Piscataway, NJ, USA). The mixtures were spun at 1300 rpm at room temperature for 10 minutes, and then the beads that contain PCR products were cleaned, denatured, and washed. Sequencing primers were annealed to the single strand DNA fragments. The sequence reaction and the detection were performed by Pyrosequencer ID (QIAGEN, Valencia, CA, USA). The results were reported as percentage of the mutation versus the wild type.

## 4. Results

### 4.1. Pathologic Findings

Intraoperative evaluation of whipple resection revealed a 3.0 cm mass in the head of the pancreas that grossly wrapped around the bile duct. Microscopic sections from the tumor showed a moderate to poorly differentiated pancreatic adenocarcinoma. The tumor cells showed both well defined gland formation and other areas with a more sheet like growth. Metastatic carcinoma was present in regional lymph nodes. Immunohistochemical stains of the adenocarcinoma involving the pancreas revealed strong expression of cytokeratin 7.

**Figure 1:**
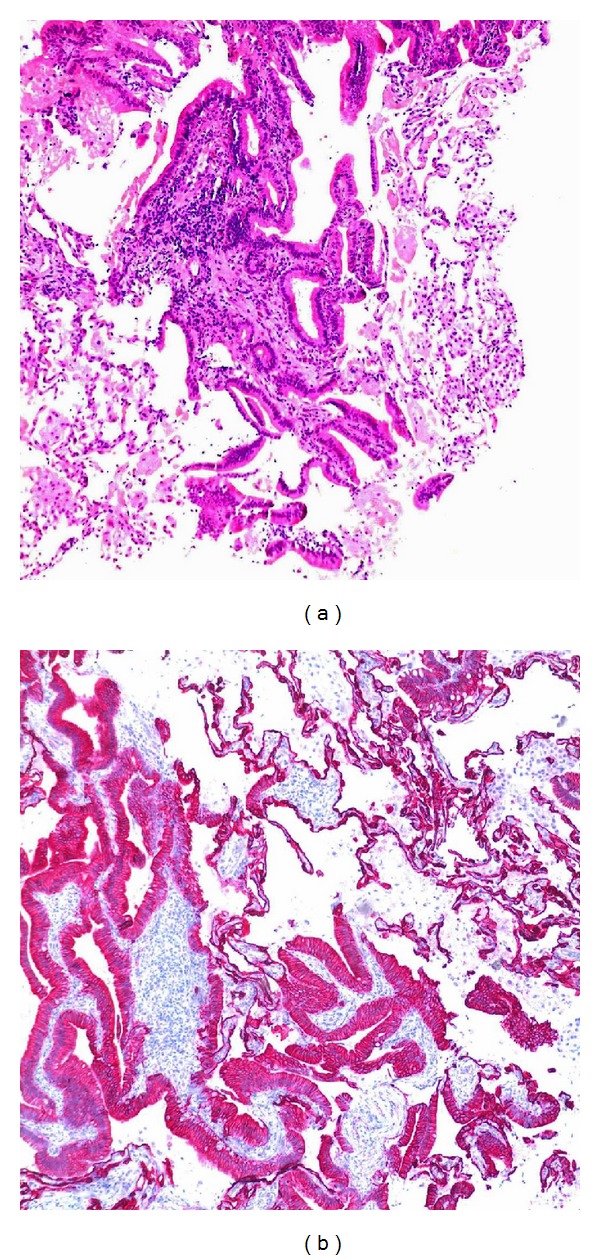
(a) H&E stain of a lung lesion showing carcinoma with lepidic growth. (b) Cytokeratin 7 immunohistochemical stain reveals strong reactivity on the lung carcinoma.

Microscopic sections of the lung biopsy showed a well-differentiated nonmucinous adenocarcinoma involving the lung with an intra-alveolar (lepidic) spread (Figure 1(a)). The hyperchromatic tumor cells were cuboidal to tall columnar with deep eosinophilic cytoplasm arranged along the alveolar walls. Necrosis was not present. Angiolymphatic invasion of tumor was not seen. The lung tumor resembled the well differentiated areas of the pancreatic tumor; however the bulk of the pancreas tumor was more poorly differentiated. The lung tumor stained strongly with cytokeratin 7 (Figure 1(b)) but was negative for cytokeratin 20 and TTF-1. On morphological and immunohistochemical grounds alone we could not definitively distinguish between a metastatic pancreatic adenocarcinoma and a nonmucinous bronchioalveolar carcinoma.

### 4.2. Pyrosequencing/Molecular Studies

Pyrosequencing of the KRAS gene in codon 12 and 13 revealed that both the pancreatic carcinoma and the lung carcinoma harbored an identical codon 12 c. 35 G > T (Gly12Val) KRAS mutation. In this clinical and pathologic context, the presence of identical KRAS mutations in the pancreas and lung was felt to be supportive of a metastatic pancreatic adenocarcinoma involving the lung.

## 5. Discussion

K-Ras is a protein encoded by the KRAS gene with GTPase activity that is involved in signal transduction of the cells and acts downstream of epidermal growth factor receptor (EGFR). It is important for cell growth and differentiation. If the KRAS gene is mutated, it becomes oncogenic leading to the activation of RAS signaling. KRAS mutations are one of the important events in the tumorigenesis of carcinomas of different organs including the pancreas, colon, and lung [3, 7, 8].

The most frequent tumor to have a KRAS mutation is pancreatic adenocarcinoma which is present in more than 90% of the cases [3]. In one study, it was shown that both primary pancreatic carcinoma and its corresponding metastasis carry the same KRAS mutation [3]. Codon 12 is the most common site for KRAS gene mutation in pancreatic adenocarcinoma with most being c. 35 G > T (Gly12Val) mutations [3]. The percentage of KRAS mutation is much lower in pulmonary adenocarcinoma (15–30%), the particular mutation seen in this case c. 35 G > T (Gly12Val) represents a subset of the KRAS mutated lung carcinomas [4–6]. Many studies have shown that KRAS mutation is more common in mucinous than nonmucinous bronchioalveolar carcinomas, especially in smokers [9, 10].

It has been shown that metastatic foci of tumor should have the same KRAS mutation as their original primary tumor [7]. However, metastatic lesions might show new mutations that were not present in the primary. In one study of colorectal adenocarcinoma, the frequency of different mutations (KRAS, BRAF, NRAS, and PIK3CA) were found to vary significantly with the location of the metastasis (lung, brain, and liver) [7]. Analysis of the mutation in the primary and metastases showed concordance in 88% of cases (86 out of 97 cases) for all 4 genes analyzed. In the discordant cases, 9 out of 11 cases had mutations that were present only in the metastatic foci.

The lungs are a common site of carcinoma metastasis. Several patterns of metastasis to the lung have been described: discrete nodules, lymphangitic carcinomatosis, tumor emboli, and the intra-alveolar (lepidic spread) pattern. The intra-alveolar pattern is the most difficult to distinguish from a primary pulmonary carcinoma—principally bronchioalveolar carcinoma (adenocarcinoma in situ). It is well known that metastases from the gastrointestinal tract will often exhibit an intra-alveolar spread [1, 2]. This can create a significant diagnostic dilemma.

Distinguishing primary lung adenocarcinoma from metastatic carcinoma is not always straight forward, and additional immunohistochemical studies can be helpful. The most useful immunohistochemical stains to differentiate gastrointestinal carcinoma from lung carcinoma are TTF-1, cytokeratin 7, cytokeratin 20, CDX2, and villin [11–14]. TTF-1 is positive in the vast majority of conventional pulmonary adenocarcinomas including nonmucinous type bronchioalveolar carcinoma (BAC). However, among mucinous BAC only 21% were positive for TTF-1 [15]. Both lung and upper gastrointestinal tract (stomach, pancreas) often express cytokeratin 7 while colorectal carcinomas are less likely to be positive. Cytokeratin 20 is expressed in most colorectal carcinomas and less frequently in upper gastrointestinal tract carcinomas. The expression of cytokeratin 20 is uncommon in conventional primary pulmonary adenocarcinoma, however, most mucinous BAC express cytokeratin 20 [11]. The transcription factor CDX2 is a reliable marker of intestinal differentiation along with the expression of villin. Among primary pulmonary adenocarcinoma however, both CDX2 and less commonly villin may be expressed in primary pulmonary adenocarcinomas particularly those with mucinous differentiation [16, 17]. Despite these markers, in many cases it may be difficult to definitively distinguish a primary from a metastatic carcinoma, particularly those with mucinous differentiation.

In this paper we demonstrate that mutational analysis of KRAS can be used diagnostically to more definitively determine the site of origin of a metastatic carcinoma—in this instance a metastatic pancreatic adenocarcinoma to the lung. Using KRAS mutation analysis as a means to support site of origin of a metastatic tumor has not been previously reported. Mutational analysis of p53 mutations were used in a study of metastatic carcinomas to the lung in which the primary site could not be identified. By comparing the p53 mutation to those present in the known primary tumor, p53 mutation analysis could correctly classify seven of nine tumors in the lung in which it was unknown if this represented a primary tumor versus a metastases [18]. A similar method was used by Nakazato et al. who used p53 mutation analysis to demonstrate that a patient with multiple lung tumors harbored both a lung carcinoma and a metastatic breast carcinoma [19].

Since the majority of pancreatic adenocarcinomas are associated with KRAS mutations and the pattern of metastasis to the lung of pancreatic carcinoma often mimics bronchioalveolar carcinoma, we feel that KRAS mutational analysis can be a useful adjunct to definitively determine the site of origin. This method is advantageous in that it can easily be used on FFPE tissue samples.

One drawback to this method is that the metastatic tumor might harbor mutations that are not present in the primary tumor [7]. Also, theoretical treatment could induce changes within the tumor or select for certain clones of tumor cells that possess a different mutation than those present in the primary tumor. Ideally, the use of KRAS mutational analysis would be most helpful if the mutation was mutually exclusive to the lung adenocarcinoma, but there also exists a possibility that the patient may develop two distinct primaries that harbor identical KRAS mutations. This method along with conventional morphologic comparison and immunohistochemistry could provide an additional level of support that the tumor represents a metastatic carcinoma. Another drawback is the requirement of having both tumor tissue from a primary tumor and the metastases for comparison. This approach should be used with caution if molecular testing is only performed on the tissue suspected to be of metastatic origin.

## 6. Conclusion

We demonstrate that the mutational analysis of KRAS can be useful to help determine the site of origin of a metastatic carcinoma to the lung in a case in which the morphological and immunohistochemical findings were not definitive. Further studies on a larger series of cases may help to validate this approach.

## References

[1] Steinke K, Süess K, Wiesner W (2000). Pulmonary metastases from pancreatic adenocarcinoma mimicking bronchoalveolar carcinoma. *European Radiology*.

[2] Rosenblatt MB, Lisa JR, Collier F (1967). Primary and metastatic bronciolo-alveolar carcinoma. *Diseases of the Chest*.

[3] Almoguera C, Shibata D, Forrester K, Martin J, Arnheim N, Perucho M (1988). Most human carcinomas of the exocrine pancreas contain mutant c-KRAS genes. *Cell*.

[4] Rodenhuis S, Slebos RJC (1992). Clinical significance of ras oncogene activation in human lung cancer. *Cancer Research*.

[5] Kobayashi T, Tsuda H, Noguchi M (1990). Association of point mutation in c-Ki-ras oncogene in lung adenocarcinoma with particular reference to cytologic subtypes. *Cancer*.

[6] Shigematsu H, Lin L, Takahashi T (2005). Clinical and biological features associated with epidermal growth factor receptor gene mutations in lung cancers. *Journal of the National Cancer Institute*.

[7] Tie J, Lipton L, Desai J (2011). KRAS mutation is associated with lung metastasis in patients with curatively resected colorectal cancer. *Clinical Cancer Research*.

[8] Tam IYS, Chung LP, Suen WS (2006). Distinct epidermal growth factor receptor and KRAS mutation patterns in non-small cell lung cancer patients with different tobacco exposure and clinicopathologic features. *Clinical Cancer Research*.

[9] Marchetti A, Buttitta F, Pellegrini S (1996). Bronchioloalveolar lung carcinomas: KRASmutations are constant events in the mucinous subtype. *Journal of Pathology*.

[10] Sakuma Y, Matsukuma S, Yoshihara M (2007). Distinctive evaluation of nonmucinous and mucinous subtypes off bronchioloalveolar carcinomas in EGFR and K-ras gene-mutation analyses for Japanese lung adenocarcinomas: confirmation of the correlations with histologic subtypes and gene mutations. *American Journal of Clinical Pathology*.

[11] Rubin BP, Skarin AT, Pisick E, Rizk M, Salgia R (2001). Use of cytokeratins 7 and 20 in determining the origin of metastatic carcinoma of unknown primary, with special emphasis on lung cancer. *European Journal of Cancer Prevention*.

[12] Marson VJ, Mazieres J, Groussard O (2004). Expression of TTF-1 and cytokeratins in primary and secondary epithelial lung tumours: correlation with histological type and grade. *Histopathology*.

[13] Barbareschi M, Murer B, Colby TV (2003). CDX-2 homeobox gene expression is a reliable marker of colorectal adenocarcinoma metastases to the lungs. *American Journal of Surgical Pathology*.

[14] Tan J, Sidhu G, Greco MA, Ballard H, Wieczorek R (1998). Villin, cytokeratin 7, and cytokeratin 20 expression in pulmonary adenocarcinoma with ultrastructural evidence of microvilli with rootlets. *Human Pathology*.

[15] Goldstein NS, Thomas M (2001). Mucinous and nonmucinous bronchioloalveolar adenocarcinomas have distinct staining patterns with thyroid transcription factor and cytokeratin 20 antibodies. *American Journal of Clinical Pathology*.

[16] Yatabe Y, Koga T, Mitsudomi T, Takahashi T (2004). CK20 expression, CDX2 expression, K-ras mutation, and goblet cell morphology in a subset of lung adenocarcinomas. *Journal of Pathology*.

[17] Mazziotta RM, Borczuk AC, Powell CA, Mansukhani M (2005). CDX2 immunostaining as a gastrointestinal marker: expression in lung carcinomas is a potential pitfall. *Applied Immunohistochemistry and Molecular Morphology*.

[18] Kandioler D, Dekan G, End A (1996). Molecular genetic differentiation between primary lung cancers and lung metastases of other tumors. *Journal of Thoracic and Cardiovascular Surgery*.

[19] Nakazato Y, Tanaka R, Seki E (2008). Differential diagnosis of primary versus metastatic pulmonary adenocarcinomas using gene mutation analyses: a case report. *Journal of Thoracic Oncology*.

