# Erratum to “The Role of KRAS Mutational Analysis to Determine the Site of Origin of Metastatic Carcinoma to the Lung: A Case Report”

**DOI:** 10.1155/2013/376018

**Published:** 2013-05-02

**Authors:** Ahmad Alkhasawneh, Hui-Jia Dong, Chen Liu, Carmen Allegra, Robert W. Allan

**Affiliations:** ^1^Department of Pathology, Immunology, and Laboratory Medicine, College of Medicine, University of Florida, P.O. Box 100275, 1600 SW Archer Road, Gainesville, FL 32610, USA; ^2^Division of Hematology and Oncology, College of Medicine, University of Florida, 1600 SW Archer Road, Gainesville, FL 32610, USA

In the paper titled “The Role of KRAS Mutational Analysis to Determine the Site of Origin of Metastatic Carcinoma to the Lung: A Case Report,” the author's name Ahmad Alkhasawneh was incorrectly listed as Ahmed N. Alkhasawneh; here it is corrected.

